# Role of Gypsum Content on the Long-Term Performance of Lime-Stabilised Soil

**DOI:** 10.3390/ma15155099

**Published:** 2022-07-22

**Authors:** Mansour Ebailila, John Kinuthia, Jonathan Oti

**Affiliations:** 1 Department of Civil Engineering, Faculty of Engineering, Bani Waleed University, Bani Waleed, Libya; 2 School of Engineering, Faculty of Computing, Engineering and Science, University of South Wales, Pontypridd CF37 1DL, UK; john.kinuthia@southwales.ac.uk (J.K.); jonathan.oti@southwales.ac.uk (J.O.)

**Keywords:** gypsum, gypseous soil, expansive soil, sulfate soil, ettringite, calcium-based stabiliser, swelling, compressive strength, linear expansion

## Abstract

The role of gypsum level on the long-term strength and expansion of soil stabilised with different lime contents is not well understood. This research, therefore, studied the effect of varying gypsum concentrations of 0, 3, 6, and 9 wt% (equivalent to the sulfate contents of 0, 1.4, 2.8, and 4.2%, respectively) on the performance of sulfate soil stabilised with two lime levels (4 and 6 wt%). This was carried out to establish the threshold level of gypsum/lime (G/L) at which the increase in G/L ratio does not affect the performance of lime-stabilised sulfate soil. Both unconfined compressive strength (UCS) and expansion, along with the derivative thermogravimetric (DTG) analysis, were adopted to accomplish the present objective. Accordingly, the result indicated that the strength and expansion were proportional to the lime and sulfate content, of which a G/L ratio of 1.5 was the optimum case scenario for UCS, and at the same time, the worst-case scenario for expansion. This discovery is vital, as it is anticipated to serve as a benchmark for future research related to the design of effective binders for suppressing the sulfate-induced expansion in lime-stabilised gypseous soil.

## 1. Introduction

Soil stabilisation by use of cementing agents is one of the conventional techniques used to obtain engineering materials having superior properties [1,2]. This technique enables enhancing the characteristics of the existing soils at the project site, thereby reaching the required specifications [3,4]. Moreover, it gives both economic and environmental advantages, as it reduces the costs associated with supplying suitable soils and landfills for unsuitable soils [5]. According to the literature, several additives such as cement and lime, among other by-product materials, have been used as stabilising agents. In this regard, the performance of cement has been comprehensively investigated in terms of different aspects including plasticity index [6], Proctor compaction [7], unconfined compressive strength [8], water absorption [9], swelling [10], and microstructure tests [11], among others. As for lime soil stabilisation, [1] investigated the effect of 3, 4, and 7 wt% of lime on the swelling of Phyllite clay and reported that 3 wt% of lime is a sufficient dosage in stabilising Phyllite clay, where the swelling was reduced up to 0%. Reference [12] proved that bentonite has a higher swelling than that of kaolin even after lime application, where 5 and 3 wt% of lime were required to suppress the swelling of bentonite and kaolin, respectively. Based on the above-mentioned studies, the main conclusion that can be drawn is that the performance of such stabilising agents depends on several factors such as soil mineralogy, binder composition, sulfate content, and moisture content, among others; thus, a proper understanding of the local conditions and an accurate soil characterisation before stabilisation, are deemed mandatory. For example, the existence of sulfate within the soil, which is commonly encountered in the form of gypsum (gypseous soils), can cause a massive expansion in the presence of calcium-based stabilisers such as cement or lime.

Gypseous soils are widespread and covered about 20% of the land in the world, with a gypsum content varying from very low (<5%) to very high (≥50%) [13,14,15]. These soils are commonly encountered in civil engineering construction, often in road pavement subgrades and layers [15]. The existence of gypseous soils in the pavement subgrades causes serious problems to the pavement, as they undergo a reversible swell–shrink behaviour [16]. The swell–shrink behaviour is partly due to the dissolution of gypsum and partly due to the expansion of soil particles upon the water flow, both of which induce volume increase and strength reduction [17,18]. Therefore, the construction of engineering structures upon such soils can be harmful, as they induce a severe challenge, threatening sustainability by inducing ground subsidence with the formation of voids, cracks, excessive settlement in dry conditions, and volume expansion upon wetting [19].

To avoid the damages associated with gypsum dissolution and soil swelling, engineers traditionally tend to use a combination of chemical and mechanical soil stabilisation techniques [15,16]. This is achieved by mixing soils with hydraulic binders such as lime or cement and compacting the stabilised mixture to achieve the maximum densification degree. However, gypseous soils, especially those having higher gypsum content, where calcium-based stabilisers are added, can undergo further excessive swelling [20,21,22]. This gypsum-induced swelling is typically associated with the formation of ettringite in a higher alkaline environment (pH ≥ 10.5) [23]. The ettringite is a highly hydrated and expansive crystalline mineral formed by the reaction of soluble sulfate (from gypsum), calcium (from lime), and alumina (from the soil) in the presence of water [24,25,26].

In the past, several research studies have identified that the onset of problems associated with ettringite formation depends on the sulfate content, lime content, and moisture content in the soil [27]. For example, reference [28] reported that the sulfate level of 2% does not harm the swelling of lime-treated soils. Aldaood [15,29] concluded that the gypsum content, beyond which the compressive strength of lime-stabilised soil is reduced, was found to be 5%. Jha and Sivapullaiah [30] recorded a gradual increase in the swelling of soil stabilised with 6 wt% of lime, as the gypsum content increases up to 6%, and such increases were more pronounced in uncured specimens. Accordingly, by and large, the main conclusion that can be drawn is that the formation of ettringite can negatively affect the swelling performance of lime-stabilised soil, and such an effect is more pronounced at a higher lime and higher sulfate content. However, research concerning the effect of lime on sulfate soil is still insufficient. For example, the threshold of the gypsum/lime (G/L) ratio, at which the increase of G/L ratio does not affect the swelling and strength performance of lime-stabilised sulfate soil, has not been scientifically established. 

Therefore, in this current study, an attempt has been made to investigate the effect of two lime levels (4 and 6 wt%) on the stabilisation of artificial sulfate kaolin soil made with varying gypsum levels (0, 3, 6, and 9 wt%). Both physical (volume change) and mechanical (change in UCS) properties have been assessed to complement the present objective in order to establish the threshold of G/L for superior swelling and strength performance. The mechanism of alteration of lime-stabilised gypseous soil was also brought out by performing a thermal analysis (TG/DTG) on both expansion and strength specimens.

## 2. Materials and Methods

### 2.1. Materials

#### 2.1.1. Kaolin

**Figure 1:**
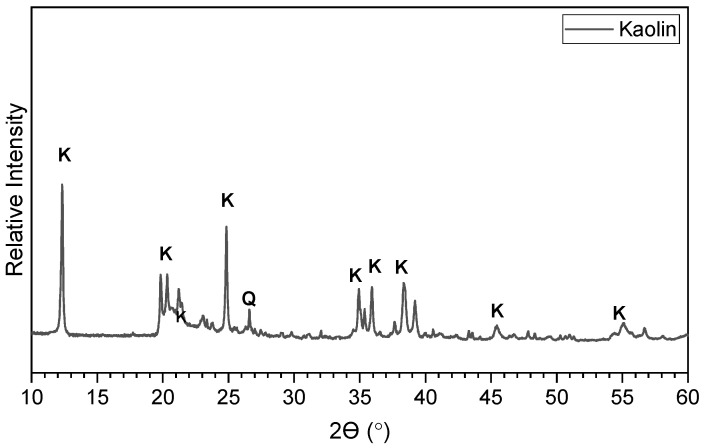
X-ray diffractogram of kaolin soil, obtained using x-ray diffraction analysis through commercial service at the University of Bath. (Note: K, kaolinite and Q, quartz).

The kaolin used in this study was semi-processed kaolin in the form of a fine powder with a pH of 5.37, a specific gravity of 2.14 ${\mathrm{mg}/m}^{3}$, and a relative density of 2.6–2.7. The kaolin used was supplied by Potterycrafts Ltd., Stoke-on-Trent, UK, under a trading name of China clay standard porcelain powder. Figure 1 shows the x-ray diffraction (XRD) curve of kaolin, which was obtained through commercial service at the University of Bath (Bath, UK). Table 1 tabulates the oxide compositions of kaolin, which were obtained using x-ray fluorescence analysis (XRF) through commercial service by Celtest Company Limited (Bangor, UK).

**Figure 2:**
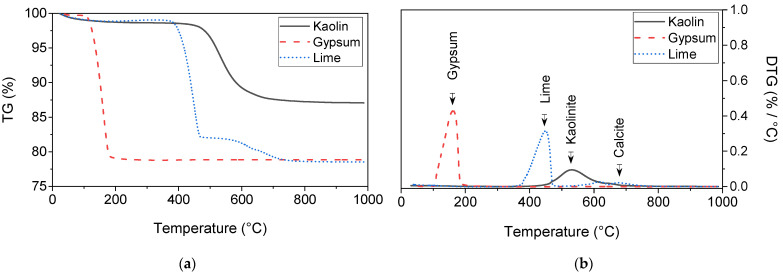
Thermogravimetric and derivative thermogravimetric (TG/DTG) curves of kaolin, lime, and gypsum: (**a**) TG and (**b**) DTG.

The mineralogical analysis revealed that its dominant minerals are kaolinite mineral and quartz, whereas the oxide composition analysis shown in Table 1 indicated that it comprised predominantly of 47.3% silicon dioxide (${\mathrm{SiO}}_{2}$) and 35.96% aluminium oxide (${\mathrm{Al}}_{2}O_{3}$). The characterisation testing results reported elsewhere [31] suggested that the kaolin used contains 27% sand, 61% silt, and 12% clay. The Atterberg limits and plasticity index analysis showed that it has a liquid limit of 56.7%, a plastic limit of 33.3%, and a plasticity index of 23.4%. Therefore, the kaolin used in this study is a medium-graded sandy silt of high plasticity. The thermogravimetric (TG) analysis shown in Figure 2 indicated that, as the temperature increases to 1000 ℃, the mass of kaolin reduced by about 10.48% in one phase. This mass loss was represented by a major endothermic peak in the DTG curve in the range of 400–700 ℃. This reduction in mass is due to the conversion of the kaolinite minerals (present in the raw kaolin) to meta-kaolinite, which is a consequence of the dehydroxylation of the lattice hydroxyl group [32,33].

#### 2.1.2. Sulfate

The sulfate type used in this study was gypsum in the physical form of a fine white powder with a percentage purity of ≥98%. It was obtained from Fisher Scientific Ltd., Loughborough, Leicestershire, Leicester, UK, under a commercial trade name of calcium sulfate dihydrate (${\mathrm{CaSO}}_{4}\cdot {2H}_{2}O$). The characterisation testing results showed that the gypsum used has a particle density (specific gravity) of 2.1 ${\mathrm{Mg}/m}^{3}$, a pH value of 7.8, and water solubility of 2 g/L (20 ℃). The TG analysis presented in Figure 2 showed that its mass was reduced by about 20% in one phase, as the temperature increased up to 1000 ℃. This mass reduction was represented in the DTG curve by a single major endothermic peak in the temperature range of 100–200 ℃, confirming its purity. The reason for the choice of using calcium sulfate (gypsum) in this study is attributed to the fact that it is the most abundant sulfate salt presented in natural sulfate-bearing soils [34,35,36,37]; thus, it was used to prepare the artificial sulfate-dosed kaolin soil.

#### 2.1.3. Lime

The lime used in this study was quicklime (CaO) in the physical form of an off-white powder with a pH of 12.6, a relative density of 3.31, a specific gravity of 2.82 ${\mathrm{Mg}/m}^{3}$, and oxide compositions as summarised in Table 1. It was supplied under a commercial trade name of Lime-base quicklime by Tarmac Cement and Lime Company, Buxton Lime and Powders, Derbyshire, Derby, UK. The TG analysis shown in Figure 2 revealed that the mass of lime decreased by about 21%, as the temperature increased to 1000 ℃. This mass reduction was represented in the DTG curve by two major endothermic peaks: one sharp peak in the range of 350–500 ℃ due to the decomposition of quicklime, and one relatively weaker peak in the range of 550–750 ℃ due to the de-carbonation of the calcite [32,33].

### 2.2. Mix Compositions

A total of eight mix compositions (see Table 2) were designed under this study to produce specimens with varying gypsum and lime contents in order to establish the gypsum/lime (G/L) threshold, at which a further increase in G/L ratio has no significant contribution to the swelling magnitude. This was achieved by batching kaolin soil with four different gypsum concentrations (0, 3, 6, and 9 wt%), and two quicklime dosages (4 and 6 wt%). The use of kaolin soil is stemmed from its consistency and homogeneity, both of which ease the understanding of the soil-binder reaction before venturing into more complex soil [38]. The chosen concentrations of gypsum are equivalent to the sulfate contents of 0, 1.4, 2.8, and 4.2% [27], representing, respectively, none, low, medium, and high levels of sulfate amount. This range of sulfate was selected based on the literature, as it is believed that the worst sulfate case scenario is within such range for a typical binder dosage of 4–to–6 wt% along with that it represents the typical range of sulfate contents found in UK soils (such as Lower oxford clay and Kimmeridge clay).

The chosen lime contents were in agreement with the typical binder range used by other researchers [17,18]. These lime contents (4 and 6 wt%) were selected among other levels such as 2 and 8 wt% in order to ensure that there would be a sufficient lime content for both cation exchange and pozzolanic reaction and, at the same time, to avoid the negative impact of a higher amount of lime on the unconfined compressive strength [39,40]. According to [41], the higher amount of lime leads to the coating of kaolin particles by a layer of adsorbed calcium, which prevents the alkaline attack and dissolution of kaolin particles [42] and, in turn, reduces the silicon and aluminium released from the soil, thereby delaying the pozzolanic reaction. In addition, [43] urged that the presence of hexagonal flaky-shaped crystals of the portlandite on the surface of soil particles induces a reduction in the cohesion between the soil particles and cementitious products.

For clarity, the sample mix code shown in Table 2 is comprised of kaolin (K), gypsum, (G) and lime (L), of which G and L are preceded by a number, which presents the material amounts. The gypsum percentage in the mix code represents the gypsum amount in the target soil material (kaolin + gypsum), while binder dosage represents the binder percentage by the mass of the target soil materials.

### 2.3. Specimen Preparation

**Figure 3:**
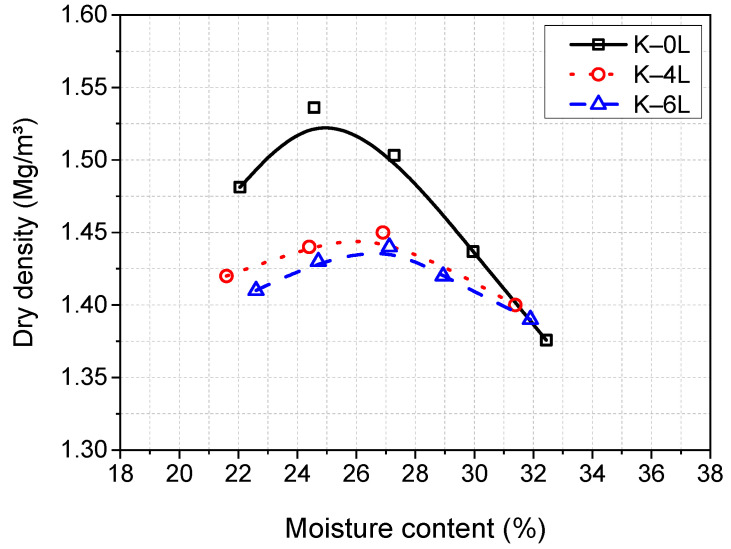
Proctor compaction curves of kaolin specimens stabilised with three different levels of lime (mainly 0, 4, and 6 wt%).

At the preliminary phase of laboratory experimentations, it was essential to establish the optimum moisture content (OMC) and maximum dry density (MDD) of stabilised soil. Therefore, proctor compaction tests were conducted in accordance with [44] to determine the OMC at which the maximum densification of specimens is obtained. Proctor compaction tests, however, were not practically possible to be performed for each material system. This was due to the time-consuming nature and cost implication of the laboratory experimentations, as well as the impracticality of establishing the OMC and MDD for each system. Hence, it was only conducted for the control mixes of K–0G–4L and K–0G–6L. The obtained results (see Figure 3) indicated that the MDD of 1455 ${\mathrm{kg}/m}^{3}$ for K–0G–4L and 1440 ${\mathrm{kg}/m}^{3}$ for K–0G–6L can be achieved at a moisture content of about 27%. However, due to the fact that soil compaction is practically carried out wet of OMC to cater for moisture losses and to actualise the best durability performance [45,46,47], a relatively higher moisture content (MC) of 30% (1.1 × OMC) was adopted for the fabrication of all the specimens.

For specimen preparation, enough dry materials (see Table 2) capable of fabricating specimen with dimensions of 100 mm in height and 50 mm in diameter were mixed in a mechanical mixer for 3 min. Thereafter, the water was introduced, and the mixing was continued for additional 3 min. Intermittent hand mixing with palette knives was also performed to remove the materials adhered to the mixing device and ensure the homogeneity of the mixture. On the completion of mixing, the semi-paste mixture was carefully accommodated into a 100 mm × 50 mm steel mould and statically compressed using a hydraulic jack as photographed elsewhere [31]. Subsequently, the specimen was extruded, weighed, wrapped in several runs of cling film, and stored in an air-tight container at a humidity of 90% and a temperature of 20 ± 2 ℃. Eventually, a total of eleven specimens were produced for each mix composition using a similar procedure. Two specimens per mix composition were used for testing the expansion while nine specimens were used for testing the unconfined compressive strength at the prescribed moist curing ages of 7, 28, and 90 days.

### 2.4. Testing Method

#### 2.4.1. Unconfined Compressive Strength (UCS)

The unconfined compressive strength test was performed on three specimens per mix proportion, in accordance with [48,49]. The test was operated using a HTE Hounsfield Testing Machine capable of exerting a load up to 10 kN and equipped with a special self-levelling device to ensure a uniaxial load application. At the end of the prescribed curing period (7, 28, and 90 days), the 100 mm ×50 mm cylindrical specimens were unwrapped, weighed and compared with their original weight at the time of casting. This comparison was carried out to discard any specimen that lost more than 2% of its original mass, complying with [48]. Thereafter, the specimens were subjected to a continuous compressive load at a strain rate of 2 mm per minute until failure, and the mean of the three values of the UCS was used as the representative UCS.

#### 2.4.2. Swelling

**Figure 4:**
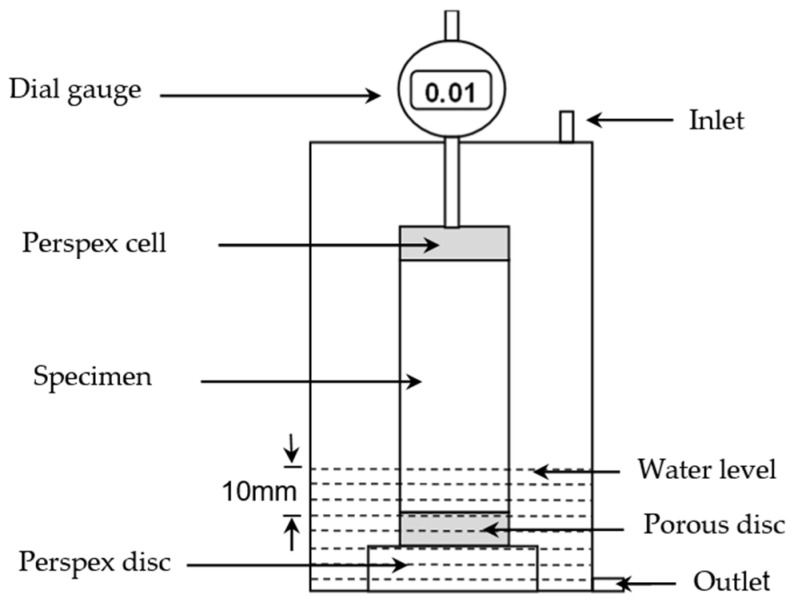
Schematic diagram of a perspex cell test set-up used for linear expansion measurement.

The degree of swelling was established by means of linear expansion measurement, in accordance with [50]. Immediately after 7 days of moist curing, about 10 mm of the top and bottom of two cylinders, per mix composition, were unwrapped by cutting the cling film using a sharp razor. The specimens were then separately placed on a porous disc located in a perspex cell as schematically presented in Figure 4. 

The perspex cells were then covered with prefabricated lids, equipped with dial gauges to measure the vertical displacement (vertical expansion) of the specimens. On the completion of the initial reading recordation, the water was carefully added to the perspex cells, through the upper inlet, using a siphon. This was performed to ensure a minimum disturbance of the compacted specimens. Followingly, the level of water was carefully increased until the exposed bottom part of the specimens was submerged in water, and such a water level was kept constant to ensure that no water evaporation from the specimens occurred. Finally, the process of soaking was monitored daily for a period of 200 days. Ultimately, the mean of the change in vertical length of the two specimens was reported as the expansion of a particular mix composition.

#### 2.4.3. Thermogravimetric and Derivative Thermogravimetric Analysis (TG/DTG)

The thermogravimetric and derivative thermogravimetric (TG and DTG) analysis was performed on randomly powdered pieces collected from the fractured UCS and swelling specimens using a TA instruments TGA55 kit, which was manufactured by Waters Ltd and obtained from TA Instruments - a subsidiary of Waters Ltd, Cheshire, UK. A test specimen weighing about 5 mg was placed in an alumina crucible, and then the test analysis was operated from room temperature up to 1000 ℃, under a flow of an argon atmosphere, at a flow rate of 25 mL/min and heating rate of 20 ℃ per minute. The representative samples used for the analysis were pre-dried in a desiccator at a temperature of 40 ℃ in aid with silica gel to ensure rapid moisture evaporation and to prevent the hydration reaction. 

## 3. Results

### 3.1. Unconfined Compressive Strength (UCS)

**Figure 5:**
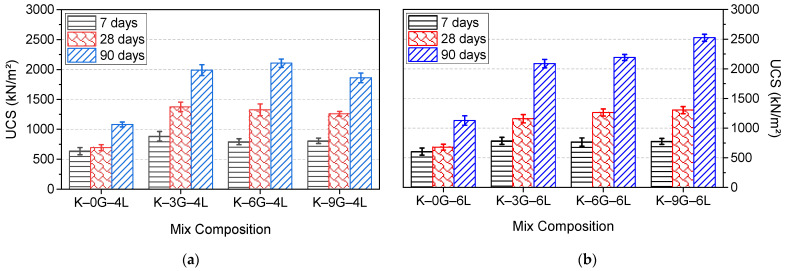
UCS development of kaolin specimens made with different gypsum concentrations (0, 3, 6, and 9 wt%) and stabilised with two different lime contents: (**a**) 4 wt% and (**b**) 6 wt%.

The unconfined compressive strength of kaolin specimens made with various gypsum amounts (0, 3, 6, and 9 wt%) and stabilised with two lime levels (4 and 6 wt%) is presented in Figure 5. The control formulations (K–0G–4L and K–0G–6L) tended to show the lowest strength at all curing ages, with the minimum strength value recorded for K–0G–4L. Additionally, the presence of varying gypsum contents in lime-stabilised kaolin improves the strength significantly. However, the extent of strength gain varies with both lime, gypsum content, as well as curing age. At 4 wt% of lime, it was observed that there was an optimum gypsum dosage shifting towards a higher gypsum dosage as the curing period increased. A lower gypsum content (3 wt%) resulted in the highest strength value up to 28 days, while a moderate (6 wt%) gypsum level led to the highest 90-day UCS. This strength trend suggests that gypsum consumption depends on the curing period. By contrast, the result of 6L–based system showed that, at all the curing ages, the UCS was gradually increased as the gypsum content increased, accounting the lowest 90-day UCS of ${2100\;\mathrm{kN}/m}^{2}$ for K–3G–6L and the greatest 90-day UCS of ${2524\;\mathrm{kN}/m}^{2}$ for K–9G–6L.

By comparing both 4L- and 6L-based series, it was evident that a mix composition of K–6G–4L (G/L = 1.5) demonstrated the highest 90-day UCS of 2105 $\mathrm{kN}/m^{2}$ at 4 wt% lime (4L), while K–6L–9G (G/L = 1.5) yielded the highest 90-day UCS of 2524 $\mathrm{kN}/m^{2}$ at 6 wt% lime (6L). This, therefore, provides convincing evidence of a consistent correlation between the UCS and G/L ratio. In the light of this information, it can be eventually summarised that the UCS is proportional to both lime and gypsum concentrations, of which a G/L ratio of 1.5 yields the highest degree of strength.

### 3.2. Linear Expansion

**Figure 6:**
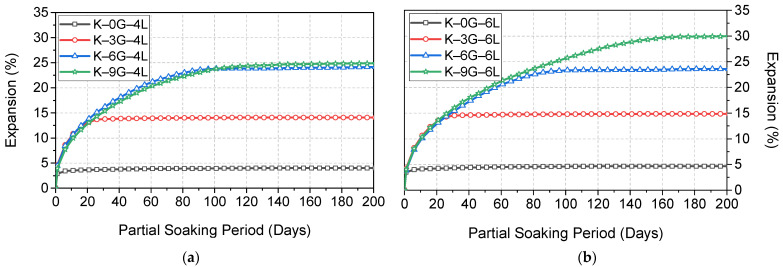
Typical expansion trends of kaolin specimens made with different gypsum concentrations (0, 3, 6, and 9 wt%) and stabilised with two different lime contents: (**a**) 4 wt% and (**b**) 6 wt%.

The typical expansion trends, over a longer soaking period of 200 days, for kaolin specimens made with different gypsum concentrations (0, 3, 6, and 9 wt%) and stabilised with two lime dosages (4 and 6 wt%), are shown in Figure 6.

Like the case of UCS, it was observed that the control formulations (without gypsum) tended to show the lowest expansion during the whole soaking period, reaching an ultimate expansion of 4% for K–0G–4L and 5% for K–0G–6L. This expansion occurred immediately after soaking in water and reached the ultimate magnitude within the first week. However, this expansion trend was not the case in the presence of gypsum, as there was a staggering expansion phenomenon, and this phenomenon was proportional to both the lime and gypsum content. In this context, the expansion (4%) of K–0G–4L was increased gradually to 14, 24.2, and 24.9%, while the expansion (5%) of K–0G–6L was increased to 14.8, 24.6, and 30%, as the gypsum increased to 3, 6, and 9 wt%, respectively. By comparing both systems (4L and 6L), it can be also inferred that there was a fixation gypsum content at which a further increase in gypsum content had no significant contribution to the ultimate expansion magnitude. This fixation gypsum dosage depended on lime content, of which 6 and 9 wt% of gypsum were the worst-case scenarios for 4 and 6 wt% lime, respectively.

### 3.3. Derivative-Thermogravimetric Analysis (DTG)

**Figure 7:**
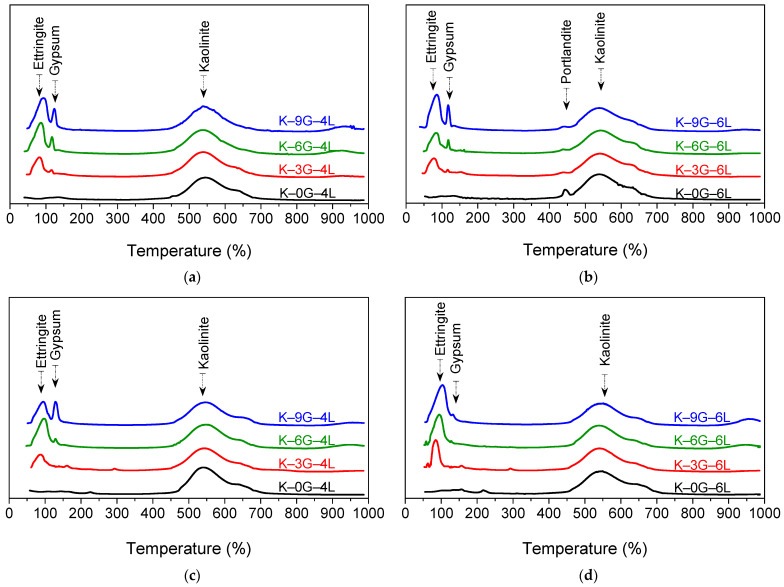
The DTG curves of kaolin specimens made with different gypsum contents (0, 3, 6, and 9 wt%) and stabilised with two lime levels (4 and 6 wt%): (**a**) kaolin specimens stabilised with 4 wt% of lime after 7 days of moist curing, (**b**) kaolin specimens stabilised with 6 wt% of lime after 7 days of moist curing, (**c**) kaolin specimens stabilised with 4 wt% of lime after 200 days of soaking, and (**d**) kaolin specimens stabilised with 6 wt% of lime after 200 days of soaking.

The DTG curves of kaolin soil specimens dosed with 0, 3, 6, and 9 wt% of gypsum and stabilised with 4 and 6 wt% of lime at two different curing conditions (7 days of moist curing and 200 days of soaking) are presented in Figure 7.

As seen in Figure 7, the main endothermic transition peaks identified in the DTG curves were: (1) ettringite peak in the temperature range of 50–100 ℃ due to the dehydration of water molecules of the ettringite; (2) gypsum peak at 100–200 ℃ due to the evaporation of the water of gypsum crystallisation and the formation of β-hemi hydrate; (3) portlandite peak at 425–475 ℃ due to the de-hydroxylation of non-consumed ${\mathrm{Ca}\;(\mathrm{OH})}_{2}$; and (4) kaolin peak at 450–700 ℃ due to decomposition of kaolinite minerals.

After 7 days of moist curing, the ettringite peak was enlarged as the gypsum content increased, and such an enlargement was more pronounced at higher gypsum and lime contents. This implies that the ettringite amount is directly proportional to gypsum and lime contents. The increasing ettringite peak was also in line with the observation of the UCS trends, suggesting valuable causativeness for the variation in strength. Furthermore, the gypsum peak was relatively invisible in the case of K–3G–4L and K–3G–6L, suggesting that 3 wt% of the gypsum can be consumed in the formation of ettringite during the 7-day moist curing period. As for the portlandite peak, it has only appeared in the case of the control mix of K–0G–6L, the existence of which indicates a sign of the uncomplete consumption of lime during the 7-day moist curing period.

Upon a prolonged (200 days) water soaking period, the DTG curves revealed considerable changes in the thermal stability of the stabilised specimens. In this regard, no trace of portlandite peak was noticed, demonstrating the importance of moisture and curing age on the completion of lime hydration. Moreover, the ettringite peak was slightly increased, relative to the unsoaked counterparts, for all the formulations, while gypsum moved contrariwise and even disappeared in some cases. At 4 wt% lime, the gypsum peak was completely disappeared in K–3G–4L and relatively invisible in the case of K–6G–4L. On the other hand, at 6 wt% lime, the gypsum peak was only detected in K–9G–6L, with a very negligible peak height. The disappearance of gypsum can be ascribed to the abundance of water molecules within the system, which tend to completely solubilise the unconsumed ingredients and act as an avenue for ion migration, hence becoming a source of ions supply at the nucleation sites of ettringite. As for the appearance of gypsum trace, this could be attributed to the excess of gypsum amount over that of the soluble sulfate threshold (gypsum/lime ratio of 1.5) consumed during the formation of ettringite.

## 4. Discussion

The above laboratory investigations brought out the influence of the variation of lime and gypsum contents on the expansion and strength performance of kaolin clay. It was observed that the strength and expansion of kaolin soil were improved on the application of lime, with more improvement being recorded for a higher lime content (6 wt%). This improvement is believed to be because of two basic reactions: short team reactions (cation exchange and flocculation/agglomeration of soil particles) and long-term reactions (pozzolanic reactions) [51,52]. When quicklime is added to soil in the presence of water, it initially hydrates (see Equation (1)), producing calcium hydroxide ($\mathrm{Ca}{\left(\mathrm{OH}\right)}_{2\;}$) and heat [52]. The hydrated lime then dissociates into divalent calcium cation (${\mathrm{Ca}}^{2+}$) and hydroxide, in accordance with the chemical reaction shown in Equation (2).

(1) $$\mathrm{CaO}+H_{2}O\;\to \;\mathrm{Ca}{\left(\mathrm{OH}\right)}_{2}+↑\mathrm{heat,}$$

(2) $$\mathrm{Ca}\;(\mathrm{OH}{)}_{2}\;\to {\mathrm{Ca}}^{2+}+{2\mathrm{OH}}^{–}$$

In the absence of sulfate, the divalent calcium cation fixes to the surface of soil particles, replacing the available exchangeable cations [17,18]. The order of cation replaceability is given by the lyotropic series of ${\mathrm{Na}}^{+}<K^{+}<{\mathrm{Mg}}^{++}<{\mathrm{Ca}}^{++}$, where higher valency cations replace those of lower valency cations [53]. This replacement in ions balances the electrostatic charges of soil particles, thereby reducing the electrochemical repulsion forces between the soil particles [54]. The reduction in repulsion forces produces a compressed double cations layer due to the change in the electronic-state charges [27,55], which therefore promotes the flocculation and agglomeration of soil particles [56]. This change in fabric decreases the clay surface area in contact with the water, thus contributing partly to the reduction of plasticity and expansion as well as the improvement of strength.

Besides the flocculation/agglomeration of soil particles, the hydroxyl ions (${\mathrm{OH}}^{–}$) are also presented [31]. The presence of ${\mathrm{OH}}^{–}$ causes a significant increase in the alkalinity (pH) value up to a range of 10–to–13 [57]. This increase in the pH value generates a corrosive environment, in which the alkaline hydrolysis of covalent bonds between Si-O and Al-O releases silicate (${\mathrm{SiO}}_{4}^{-4}$) and aluminate ($\mathrm{Al}{\left[\mathrm{OH}\right]}_{4}^{-}$) [58]. The rate at which these components are released depends on several factors. These factors include (1) the soil mineralogy, in which montmorillonite provides the greatest dissolution rate; (2) the water amount, as it acts as an avenue for ion migration; and (3) the bond of the soil sheets, of which the octahedral sheet possesses a relatively weaker Al-O bond relative to the tetrahedral sheet (Si-O) [41].

Following the dissolution of clay minerals, the pozzolanic reactions thereafter take place between the excess lime and dissolved (Al and Si) ions from treated soil [59]. These pozzolanic reactions produce different hydrated compounds such as calcium-silicate hydrate (C–S–H) and calcium aluminate hydrate (C–A–H) [57,60]. These hydrated products are time and lime content-dependent [15,16]; thus, the strength increases as the curing age and the lime content increases [13]. The hydrated products then crystallise with time and bind the soil particles by infilling the inter-aggregate pore space [53]. This action, therefore, forms a stiff matrix and induces pore-blocking and system densification, all of which improve the swelling behaviour and the mechanical strength of soil [58].

In the presence of gypsum, however, an alteration in the short-term reactions (cation exchange and flocculation/agglomeration of soil particles) and long-term reactions (pozzolanic reactions) can occur due to the dissolution of gypsum [61]. This is due to the fact that the dissolution of gypsum in water releases sulfate ions, which accordingly react with the alumina (released from the soil) and calcium (released from lime) to form ettringite [56]. The ettringite is a highly hydrated and expansive crystalline mineral typically growing within soil pores in the form of a needle-like structure with a higher surface area and unsatisfied charge [24,25,26]. According to Beetham et al. [58] and Puppala [62], the formation of ettringite in lime-based systems takes place as shown in Equation (3).

(3) $${6\mathrm{Ca}}^{2+}{+2\mathrm{Al}(\mathrm{OH})}_{4}^{–}{+4\mathrm{OH}}^{–}+3{\left({\mathrm{SO}}_{4}\right)}^{2–}{+26H}_{2}O\;\to {\;\mathrm{Ca}}_{6}{\left[{\mathrm{Al}(\mathrm{OH})}_{6}\right]}_{2}\;\cdot \;{\left({\mathrm{SO}}_{4}\right)}_{3}\;\cdot {\;26H}_{2}O$$

Under the sealed condition (moist curing condition), the growth of ettringite is controlled due to the lower dissolution ratio of gypsum and the unavailability of enough water for dissolving gypsum [61]. Therefore, the amount of ettringite formed under the moisture-curing condition (UCS specimens) could be accommodated within the matrix pores [16]. The growth of ettringite in pores under the sealed condition is also beneficial as it (1) reduces the porosity, due to its growth within the pores, (2) interlocks the system, due to its growth around the soil particles, and (3) dewaters the system, due to its high-water absorption capability [30], all of which contribute to a higher strength degree. This is, therefore, in support of why the compressive strength increased corresponding to the increase of gypsum content under this study.

However, under the soaking condition, the movement of water within the stabilised soil tends to solubilise the unconsumed ingredients and acts as an avenue for ion migration, facilitating the diffusion of reactants [58]. Therefore, the water becomes a source of ions supply at the nucleation sites of ettringite [31,61]. This then results in the formation of extensive quantities of ettringite [58], which then continue to grow due to its higher water absorption capacity. The extensive growth of ettringite is not beneficial, as it cannot be accommodated within the system pores [62], thereby causing a drastic expansion. According to the result of this study, the increase in expansion was, however, not in linear proportion with the increase in gypsum content, in which there was a gypsum threshold, beyond which a further increase of sulfate content has no significant contribution to the expansion. This threshold of gypsum dose was also dependent on the lime content, of which 6 and 9 wt% of gypsum were the worst-case scenarios for 4 and 6 wt% of lime, respectively. Based on the findings of DTG, this difference in the expansion can be associated with the amount of the ettringite formed, where the ettringite peak in both DTG curves was in line with the expansion trends.

Overall, the result of the laboratory experimentations reported in this paper suggests that the strength and expansion performance of lime-stabilised kaolin were proportional to gypsum (G) and lime (L) content, of which a G/L ratio of 1.5 was the optimal case scenario for the UCS and the worst-case scenario for the expansion. This is of great importance and practically pertinent to all the civil, geotechnical, and geological engineers/researchers involved in the development of an effective binder. This discovery is vital, as it is anticipated to serve as a benchmark for future research related to the design of effective binders for suppressing the sulfate-induced expansion in lime-stabilised sulfate soil.

## 5. Conclusions

The main conclusions emerging from the results of the laboratory experimentations reported in this study can be drawn as follows:

- The addition of lime to kaolin soil in the absence of gypsum increases the compressive strength and reduces the expansion through the change of fabric (cation exchange, flocculation/agglomeration of soil particles) and the formation of pozzolanic reactions. However, in the presence of sulfate, lime can yield a higher strength magnitude, but it also induces a drastic expansion due to the formation of an excessive ettringite.
- The consumption of gypsum during the nucleation and growth of ettringite in lime-stabilised gypsum-bearing soil is a function of lime content, moisture content, and curing condition.
- The presence of sulfate (gypsum) increases the compressive strength and the expansion of lime-stabilised soil as the gypsum content increases, with a more pronounced effect at a higher gypsum content and higher lime content. This is because the amount of formed ettringite is a function of both lime and gypsum contents. Under the sealed condition, the ettringite improves the strength through the reduction of porosity and interlocking of the matrix, while under the water soaking condition, it causes expansion due to its higher water absorption capability.
- The strength and expansion performance of lime-stabilised soil were directly proportional to the gypsum (G) and lime (L) content, of which a G/L ratio of 1.5 yielded the optimum condition for the UCS and the worst-case scenario for the expansion.

## Figures and Tables

**Table 1:** Oxide compositions of kaolin and lime, obtained using x-ray fluorescence analysis (XRF) through commercial service by Celtest Company Limited.

Oxide Compositions (%)		Kaolin	Lime
Calcium oxide	CaO	<0.01	71.56
Magnesium oxide	MgO	0.21	0.58
Silicon dioxide	${\mathrm{SiO}}_{2}$	47.32	0.67
Aluminium oxide	${\mathrm{Al}}_{2}O_{3}$	35.96	0.07
Sodium oxide	${\mathrm{Na}}_{2}O$	0.07	<0.02
Phosphorus pentoxide	$P_{2}O_{5}$	0.12	0.03
Iron or ferric oxide	${\mathrm{Fe}}_{2}O_{3}$	0.69	0.05
Manganese oxide	${\mathrm{Mn}}_{2}O_{3}$	0.02	0.02
Potassium oxide	$K_{2}O$	1.8	<0.01
Titanium dioxide	${\mathrm{TiO}}_{2}$	0.02	<0.01
Vanadium oxide	$V_{2}O_{5}$	<0.01	0.02
Barium oxide	BaO	0.07	<0.01
Acid soluble sulfate	${\mathrm{SO}}_{3}$	0.01	0.19
Loss on Ignition	LOI	13.1	27.4

**Table 2:** Mix compositions of artificial sulfate-dosed kaolin specimens stabilised with two different lime dosages (4 and 6 wt%).

Mix Code	Mix Compositions (wt%)			MDD ^1^	MC ^2^	Mix Ingredients (g) per Specimen			
	Target Soil Material		Lime			Kaolin	Gypsum	Lime	Water
	Kaolin	Gypsum							
K–0G–4L	100	0	4	30	1455	288.40	0.00	11.54	89.98
K–3G–4L	97	3	4	30	1455	279.75	8.65	11.54	89.98
K–6G–4L	94	6	4	30	1455	271.10	17.30	11.54	89.98
K–9G–4L	91	9	4	30	1455	263.20	25.20	11.54	89.98
K–0G–6L	100	0	6	30	1440	280.10	0.00	16.81	89.07
K–3G–6L	97	3	6	30	1440	271.70	8.40	16.81	89.07
K–6G–6L	94	6	6	30	1440	263.30	16.80	16.81	89.07
K–9G–6L	91	9	6	30	1440	254.90	25.20	16.81	89.07

^1^ Maximum dry density (${\mathrm{kg}/m}^{3}$). ^2^ Moisture content (%).

## Data Availability

Not applicable.
